# Highly accurate component placement does not improve patient‐reported outcomes after total hip arthroplasty: A computerised tomography‐based study

**DOI:** 10.1002/jeo2.70722

**Published:** 2026-04-16

**Authors:** Raymond Guntae Kim, Lucy Jane Salmon, Gerard Smith, Michael Dudley O'Sullivan

**Affiliations:** ^1^ North Sydney Orthopaedic Research Group, The Mater Clinic Wollstonecraft New South Wales Australia; ^2^ Faculty of Medicine and Health, Sydney Medical School The University of Sydney Camperdown New South Wales Australia; ^3^ North Sydney Orthopaedic and Sports Medicine Centre, The Mater Clinic Wollstonecraft New South Wales Australia; ^4^ School of Medicine, Sydney Campus The University of Notre Dame Australia Darlinghurst New South Wales Australia; ^5^ Corin Group Pymble New South Wales Australia

**Keywords:** accuracy, anteversion, inclination, navigation, patient reported outcome measures, total hip arthroplasty

## Abstract

**Purpose:**

To compare total hip arthroplasty (THA) outcomes between those with and without highly accurate component placement relative to the preoperative plan on computerised tomography (CT) criteria.

**Methods:**

Primary THA patients with preoperative and postoperative CT scans who completed baseline and 1‐year patient‐reported outcomes (PROMs) were included. Patients were allocated to the ‘precise anatomic restoration (PAR)’ group if they met all criteria for component position relative to the preoperative plan: within 5 mm of femoral offset and leg length and within 10 degrees of combined anteversion and inclination of the acetabular cup. Those who did not meet these criteria were allocated to the control group. Outcomes were compared between groups for 1‐year PROMs, including the Oxford hip score (OHS), hip dysfunction and Osteoarthritis Outcome Score for Joint Replacement Score (HOOS JR) and satisfaction.

**Results:**

Of 125 participants, 70 (56%) were allocated to PAR while 55 (44%) to control. There was no significant difference between groups for 12‐month OHS (*p* = 0.438), HOOS JR (*p* = 0.630), the proportion achieving PASS for OHS (*p* = 0.495), HOOS JR (*p* = 0.575) or satisfaction (*p* = 0.854). There was no significant correlation between component position error on CT and 12‐month PROMs.

**Conclusion:**

Highly accurate THA component placement does not translate to superior PROMS, compared to those with small errors in component placement at 1 year after THA. The implications for hip arthroplasty practice are substantial: achieving high levels of technical accuracy through advanced technologies may not consistently translate into measurable improvements in patient outcomes.

**Level of Evidence:**

Level II.

**Abbreviations**
3D: three‐dimensional
APP: anterior pelvic plane
COR: centre of rotation
CT: computerised tomography
HOOS JR: Hip dysfunction and Osteoarthritis Outcome Score for Joint Replacement Score
LLD: leg length discrepancy
MIC: Minimal Clinically Important Change
OA: osteoarthritis
OHS: Oxford Hip Score
PAR: precise anatomic restoration
PASS: Patient Acceptable Symptom State
PROMs: patient reported outcomes
PSI: patient‐specific instrumentation
SD: standard deviation
THA: total hip arthroplasty
VAS: visual analogue score

## INTRODUCTION

Successful total hip arthroplasty (THA) is dependent on a variety of factors including appropriate patient selection, avoiding complications, correct preoperative templating and accomplishing those targets [22]. Accurate component placement of both the acetabular cup and femoral stem reduces the risk of impingement, edge loading, implant wear, leg‐length discrepancy and instability, which can lead to heightened rates of revision [11].

Modern preoperative templating techniques use three‐dimensional (3D) virtual software systems to calculate the biomechanical boundaries of both the acetabular and femoral components during motion, thus allowing optimal virtual selection of implant size and placement [5]. To date, numerous methods to achieve accurate components placement in THA exist, including robotic‐assisted surgery, navigated technology and patient‐specific instrumentation (PSI), which reduce rate of dislocations and revisions when compared to manual techniques [1].

The Optimized Positioning System^TM^ (OPSInsight^TM^, Corin Ltd.) is a 3D preoperative planning platform that calculates alignment to optimise hip biomechanics during functional activities, and produces 3D‐printed PSI cutting guides that are used with a laser‐navigation system to guide component placement accurately [27, 28]. While an improvement in accuracy has been demonstrated with the increased adoption of navigated technology in THA [1, 5, 27, 28], there is a paucity of literature on the implications of improved accuracy, as it relates to patient‐reported outcome measures (PROMs). A recent study demonstrated acetabular component position and femoral offset change relative to the native state, assessed with radiographs was associated superior PROMS after THA [25]. However, when compared to standard radiographs, assessment of component position is considerably more accurate with computerised tomography (CT) [7, 22], and the relationship between PROMS and component position assessed with CT has not been reported.

The aim of this case‐control study was to investigate whether accurate THA component placement relative to a preoperative plan (precise anatomic restoration [PAR] group) was associated with superior PROMs at 12 months, compared to those who did not have accurate component THA placement (controls) on CT criteria. The null hypothesis was there would be no difference in outcomes between the two groups.

## METHODS

A retrospective analysis of a prospective database of consecutive patients who underwent elective THA by a single surgeon between July 2019 and June 2020 was performed. The database has approval from St Vincent's Human Research Ethics Committee, Sydney REF 2019/ETH02851, and all subjects provided informed consent. Eligible patients met the inclusion criteria: primary THA under the care of a single surgeon, consent to participate in the institutional arthroplasty outcomes registry, baseline PROMs, pre and postoperative CT imaging (*n* = 129). Exclusion criteria were patients who did not complete PROMs at 12 months (*n* = 4).

### Preoperative templating

All patients had preoperative templating with the OPSInsight^TM^ 3D preoperative planning platform, which includes an anteroposterior radiograph of the pelvis, three functional lateral radiographs (standing, flexed seated and standing with 90 degree flexion of the contralateral hip) and a low‐dose CT scan [9, 10]. Similar to the work by Spencer‐Gardner et al. [27], the senior surgeon made adjustments as necessary to the draft template produced by engineers to restore combined offset and leg length as well as calculate the optimal acetabular and femoral component placement to prevent impingement, edge‐loading and dislocation. Changes to the version were virtually templated in the computer software using a ratio previously described by Lembeck et al. [14] A 3D‐printed PSI guide was manufactured utilising the preoperative CT scan.

### Surgical technique

THA was performed utilising a standard posterolateral approach and a 3D‐printed PSI. All patients received a Trinity cup, and the stem was Corail in 103, Metafix in 5 and Taperfit in 17. All patients received a cementless acetabular cup and of these, 108 (86%) patients received a cementless femoral stem. A femoral neck osteotomy was performed, using standard landmarks, at a predetermined level. Following excision of the femoral head, the acetabulum was exposed, and the 3D‐printed PSI guide was placed into the acetabulum utilising bony landmarks in accordance with the preoperative CT scan. A handle for the acetabular guide projected a laser beam onto the ceiling, and this was matched with another target laser beam mounted on the pelvis to account for intraoperative movements of the pelvis [27]. Reaming and placement of the acetabular component was undertaken by aligning the laser from the handle with the laser target already on the ceiling. A leg length and offset guide was utilised intraoperatively in addition to clinical assessment of leg length, ROM and stability.

### Postoperative radiological measurements

A single 3D model of the hip joint including the femoral and acetabular implants was created by overlaying the pre and postoperative CT scans utilising computer software. For leg length and offset, 3D models of implanted acetabular cup and femoral stem were compared to pre‐operative reference images in the overlay to determine the changes to the centre of rotation (COR) of the femur and of the acetabulum. To measure acetabular cup radiographic definitions described by Murray [18] were utilised and referenced against the anterior pelvic plane in the overlay image, with a reference to the posterior condylar axis for stem anteversion.

### PROMs

Subjects completed a series of PROMs preoperatively and 12 months after THA including the OHS, hip dysfunction and Osteoarthritis Outcome Score for Joint Replacement Score (HOOS JR) Score, measures of general health with the EuroQol (EQ)‐5D and visual analogue score (VAS) for hip pain. After THA level of satisfaction (very satisfied, satisfied, neutral, dissatisfied/very dissatisfied), whether they experienced improvement compared to preoperative state (much better/a little better/about the same/a little worse/much worse) and whether they would have the same surgery again was also assessed. All data were collected and managed using REDcap electronic data capture tools [8].

### Group allocation

Patients were allocated to PAR if they met all the following post‐operative CT criteria relative to the preoperative plan: within 5 mm of femoral offset, within 5 mm of leg length, within 10 degrees of acetabular inclination and within 10 degrees of combined acetabular anteversion. All other patients were allocated to the control group. Variation of 5 mm femoral offset and leg length was chosen based on previously published literature demonstrating reduced abductor strength and alterations in gait kinematics [17, 20]. Variation in 10 degrees was chosen due to the minimum clinically important difference reported in literature based on 2D templated ‘safe‐zone’ acetabular cup targets of 40 degrees of inclination and 20 degrees of anteversion [15]. Combined anteversion was defined as per Dorr et al. [3].

### Statistical analysis

Statistical analysis was undertaken using SPSS v. 27.0 (IBM, USA). Normally distributed continuous variables are described with mean and standard deviation (SD), and comparison between groups was performed with independent *t*‐tests. If normality was violated, nonparametric descriptive data is reported, and comparison between groups was performed with Mann–Whitney *U*‐test. The minimal clinically important change (MIC) was defined using anchor‐based methods as eight points for the Oxford Hip Score (OHS) [6] and 18 points for the HOOS JR Score [16]. With 70 PAR and 55 control participants, the study had 80% power (*α* = 0.05) to detect differences of approximately 6 points for HOOS‐JR and three points for OHS—well below the established MICs. Dichotomised elements of the EQ‐5D were compared using the Chi‐square test. Pearson correlation coefficient determined associations between radiological elements of PAR and PROMs. With 125 participants, the study had 80% power (*α* = 0.05) to detect small to moderate correlations (*r* ≥ 0.20). Component positioning error was visualised using bivariate scatter plots. Inclination error was plotted against combined anteversion error to represent combined cup orientation, while offset error was plotted against leg length error to represent femoral reconstruction. These paired parameters were displayed together because their biomechanical effects are interdependent. Each point represents an individual case, with colour denoting the OHS. The Patient Acceptable Symptom State (PASS) threshold was defined using anchor‐based methods for the OHS was 40 [6], and HOOS JR score was 77 [12]. Analyses were prespecified and focused on a limited number of clinically relevant outcomes; therefore, no formal correction for multiple testing was applied. Statistical significance was defined as *p* < 0.05.

## RESULTS

There were 129 patients who met the inclusion criteria, of these 4 were excluded from the study as they did not complete 12‐month PROMs, leaving 125 (97%) for final analysis with a mean age of 66 years.

The proportion of participants who met the individual PAR criteria is shown in Table 1. Overall, high levels of accuracy were achieved using the 3D‐printed PSI guide to the planned target. The mean component placement error (difference from planned) was 3.6° (SD 5.4) for acetabular cup anteversion, 3.6° (SD 4.8) for acetabular cup inclination, 0.9° (SD 7.4) for combined anteversion, 1.5 mm (SD 3.7) for leg length and 0.6 mm (SD 3.5) for offset.

**Table 1:** Precise anatomic reconstruction (PAR) criteria.

Component	PAR criteria	*N* meeting PAR criteria of 125 (%)
Acetabular cup inclination	Within 10 degrees	115 (92)
Combined anteversion	Within 10 degrees	105 (84)
Leg length	Within 5 mm	102 (82)
Offset	Within 5 mm	109 (87)

Abbreviation: PAR, precise anatomic restoration.

A total of 70 (56%) fulfilled all PAR criteria and were allocated to the PAR group, and 55 (44%) were allocated control group. The demographics and baseline variables for the two groups are shown in Table 2. There was a statistically significant higher proportion of patients who had osteoarthritis (OA) in the PAR group compared to the control group (*p* = 0.005). There were no significant differences in baseline PROMs between groups.

**Table 2:** Demographics and baseline variables for the PAR and control groups.

Baseline variable	PAR group (*n* = 70)	Control (*n* = 55)	*p*‐value
Mean age years (SD)	66.3 (9)	65.2 (10)	0.528
BMI 30 or more kg/m^2^ *N* (%)	15 (21)	20 (36)	0.065
Diagnosis *N* (%)			
Osteoarthritis	69 (99)	47 (86)	0.043
Avascular necrosis	‐	2 (4)	
Developmental dysplasia	1 (1)	5 (9)	
Inflammatory arthritis	‐	1 (2)	
Male *N* (%)	38 (54)	27 (49)	0.564
Median hip pain (IQR)	60 (28)	65 (27)	0.237
Median HOOS JR score (IQR)	59 (14)	56 (17)	0.404
Median Oxford score (IQR)	25 (11)	23 (17)	0.365
EQ‐5D no problems with mobility	7 (10%)	6 (11%)	0.841
EQ‐5D no depression or anxiety	32 (46%)	23 (43%)	0.729

Abbreviations: BMI, body mass index; EQ, EuroQol; HOOS JR, hip dysfunction and Osteoarthritis Outcome Score for Joint Replacement score; IQR, interquartile range; PAR, precise anatomic restoration; SD, standard deviation.

**Figure 1:**
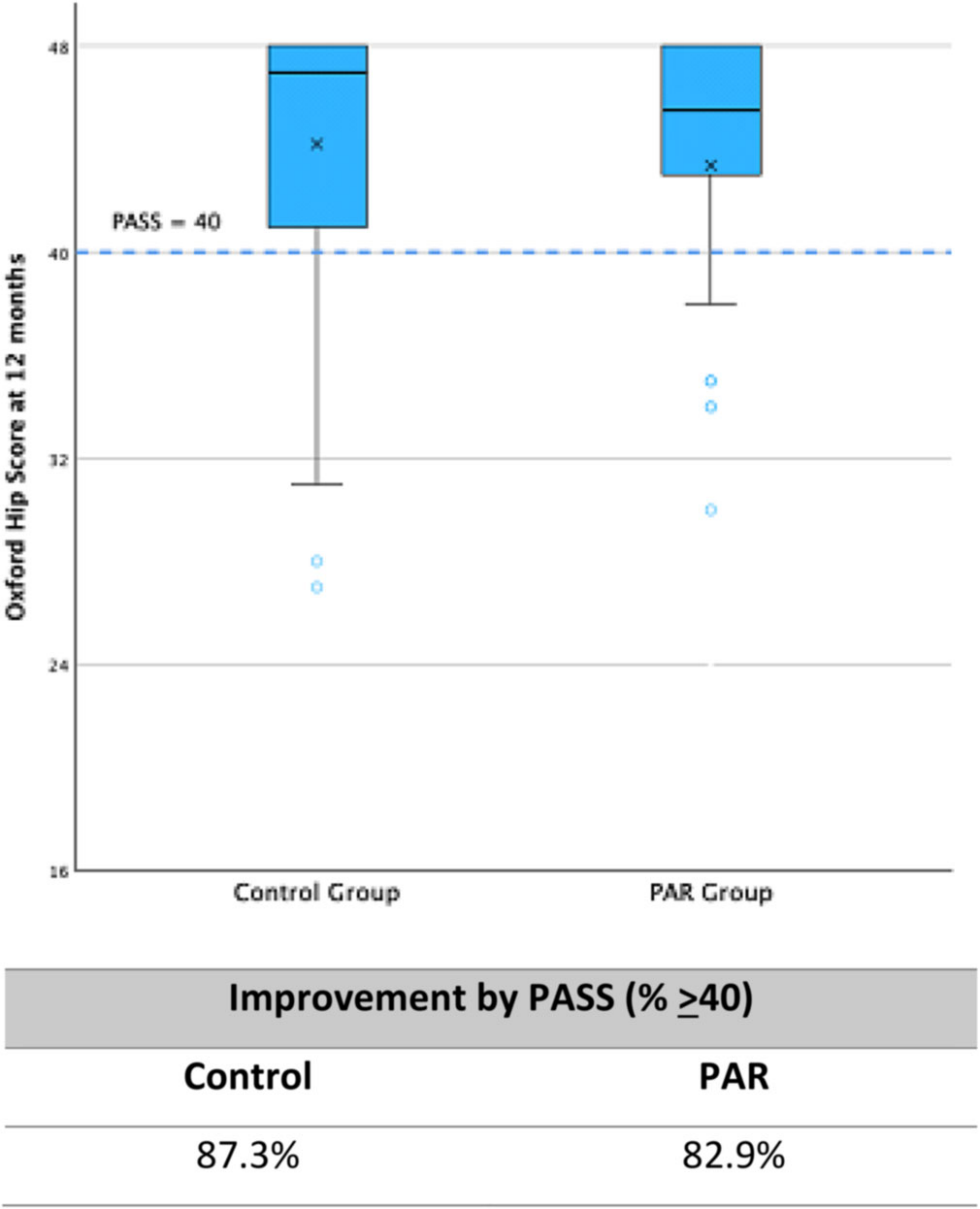
Boxplot of the Oxford hip score at 12 months for the control group and the PAR group. PAR, precise anatomic restoration; PASS, Patient Acceptable Symptom State.

The 1‐year PROMs for the PAR and Control Group are shown in Table 3. There was no significant difference between groups for 12‐month PROMs including the OHS (Figure 1), HOOS JR score, VAS score for pain and EQ‐5D, or rates of satisfaction.

**Table 3:** 1‐Year patient‐reported outcomes of the PAR and control groups.

1‐Year PROMs	PAR Group (*n* = 70)	Control (*n* = 55)	*p*‐value
Median Hip Pain VAS/100 (IQR)	2.0 (8)	0 (9)	0.443
Median HOOS JR Score (IQR)	96 (19)	92 (19)	0.888
>PASS of 77 on HOOS JR *n* (%)	60 (86%)	49 (89%)	0.575
Change in HOOS JR > MIC 18 *n* (%)	45 (83%)	55 (79%)	0.506
Median Oxford hip score (IQR)	46 (5)	47 (7)	0.295
>PASS of 40 on Oxford *n* (%)	58 (83%)	48 (87%)	0.495
Change in Oxford >MIC 8 *n* (%)	51 (93%)	63 (90%)	0.593
EQ mobility no problem *n* (%)	54 (77%)	44 (80%)	0.700
Satisfied or very satisfied *n* (%)	67 (96%)	53 (96%)	0.854
Improvement—Better than preoperative *n* (%)	67 (96%)	55 (100%)	0.299
Same surgery again *n* (%)	66 (94%)	51 (93%)	0.526

Abbreviations: EQ, EuroQol; HOOS JR, hip dysfunction and Osteoarthritis Outcome Score for Joint Replacement Score; IQR, interquartile range; MIC, minimally important change; PAR, precise anatomic restoration; PASS, Patient Acceptable Symptom State; VAS, visual analogue score.

**Figure 2:**
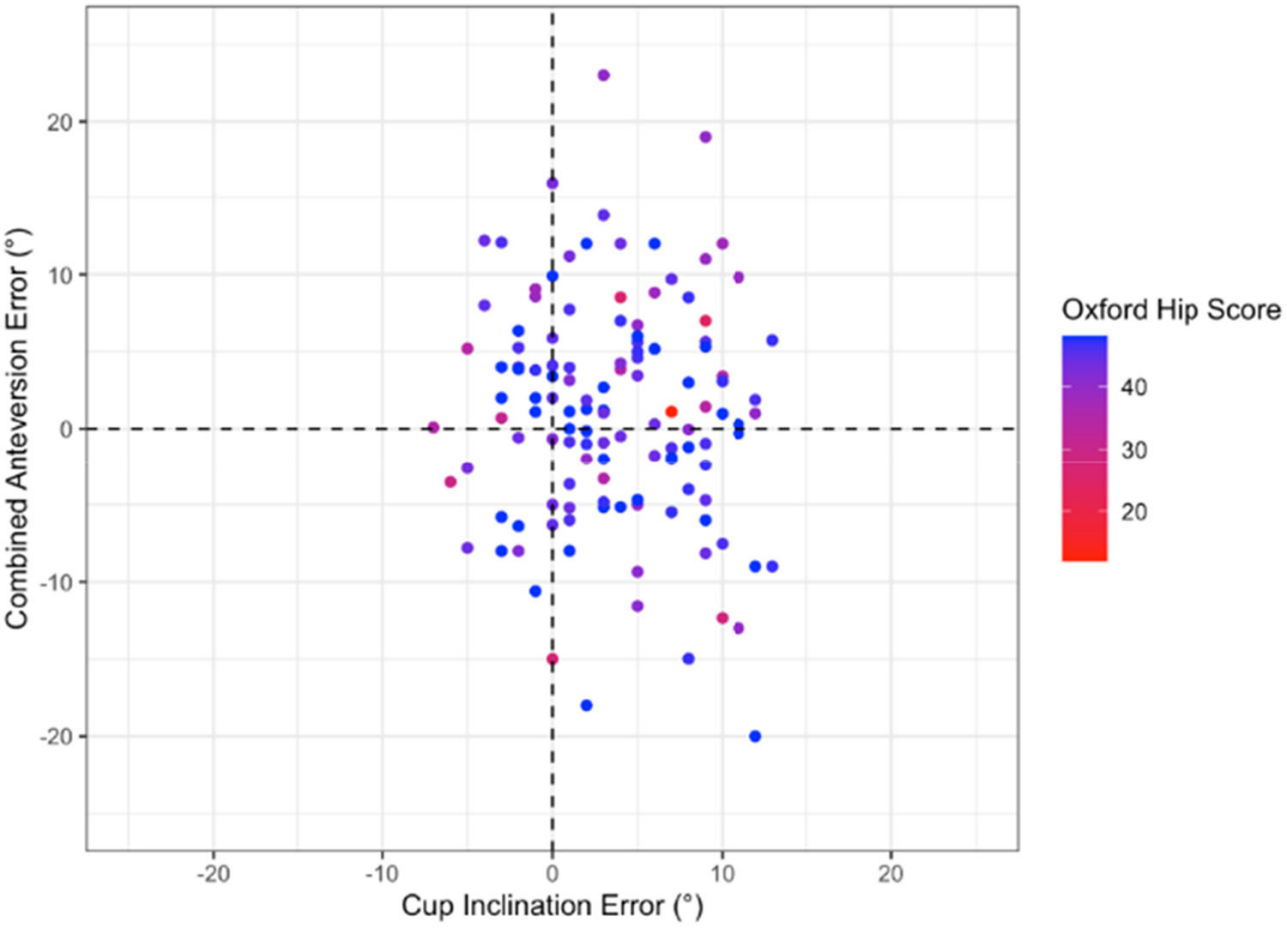
Distribution of Oxford hip score according to acetabular cup inclination error and anteversion error (Figure 2) and leg length error and offset error (Figure 3). Cup inclination and anteversion are displayed together to represent the combined acetabular orientation. Progression from red to blue representing higher (better) Oxford scores.

**Figure 3:**
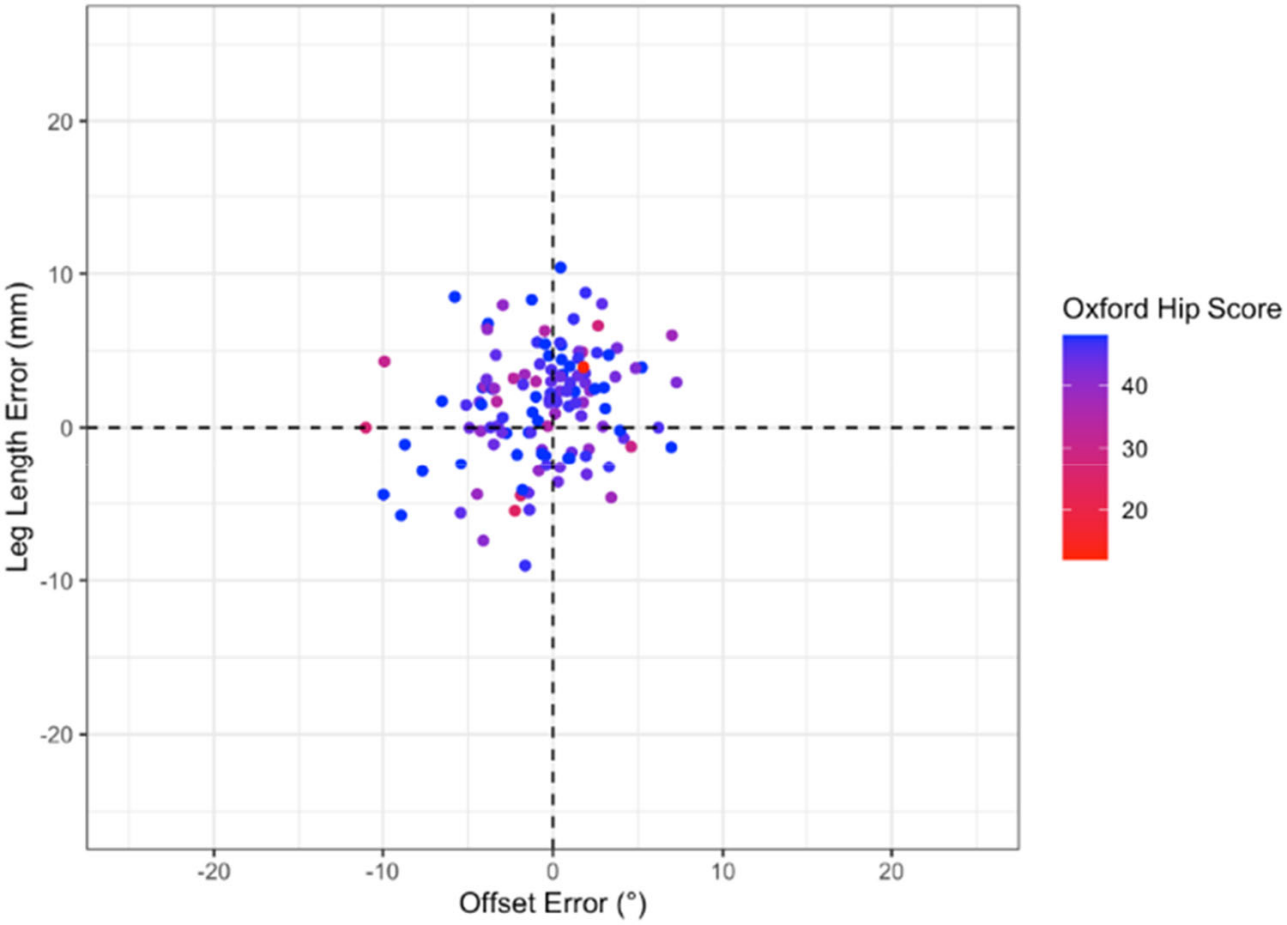
Distribution of Oxford hip score according to acetabular cup inclination error and anteversion error (Figure 2) and leg length error and offset error (Figure 3). Cup inclination and anteversion are displayed together to represent the combined acetabular orientation. Progression from red to blue representing higher (better) Oxford scores.

**Figure 4:**
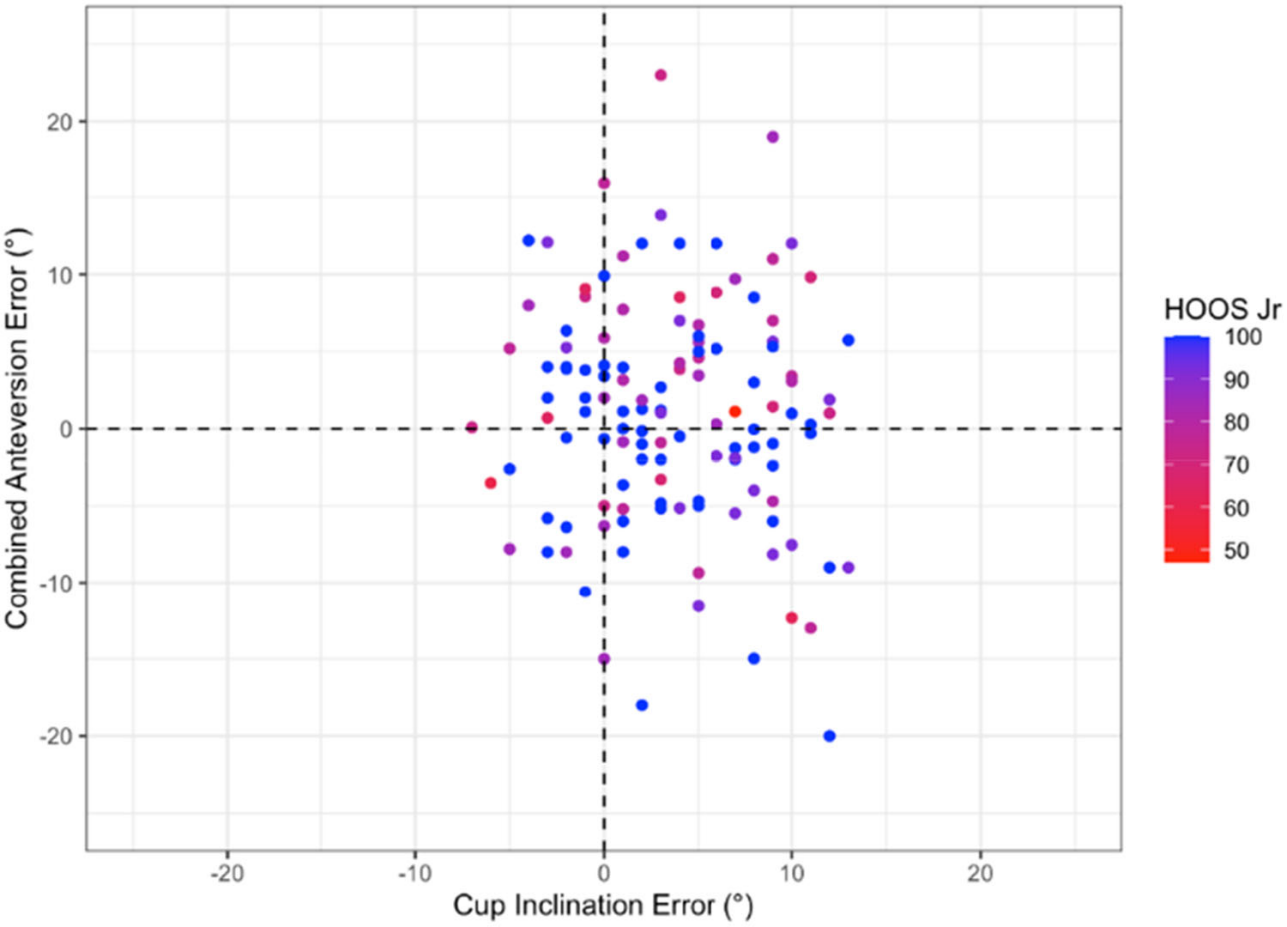
Distribution of HOOS JR according to acetabular cup inclination error and anteversion error (Figure 4) and leg length error and offset error (Figure 5). Leg length and femoral offset are displayed together to reflect their combined influence on hip biomechanics and functional limb symmetry. Progression from red to blue representing higher (better) Oxford Scores. HOOS JR, hip dysfunction and Osteoarthritis Outcome Score for Joint Replacement Score.

**Figure 5:**
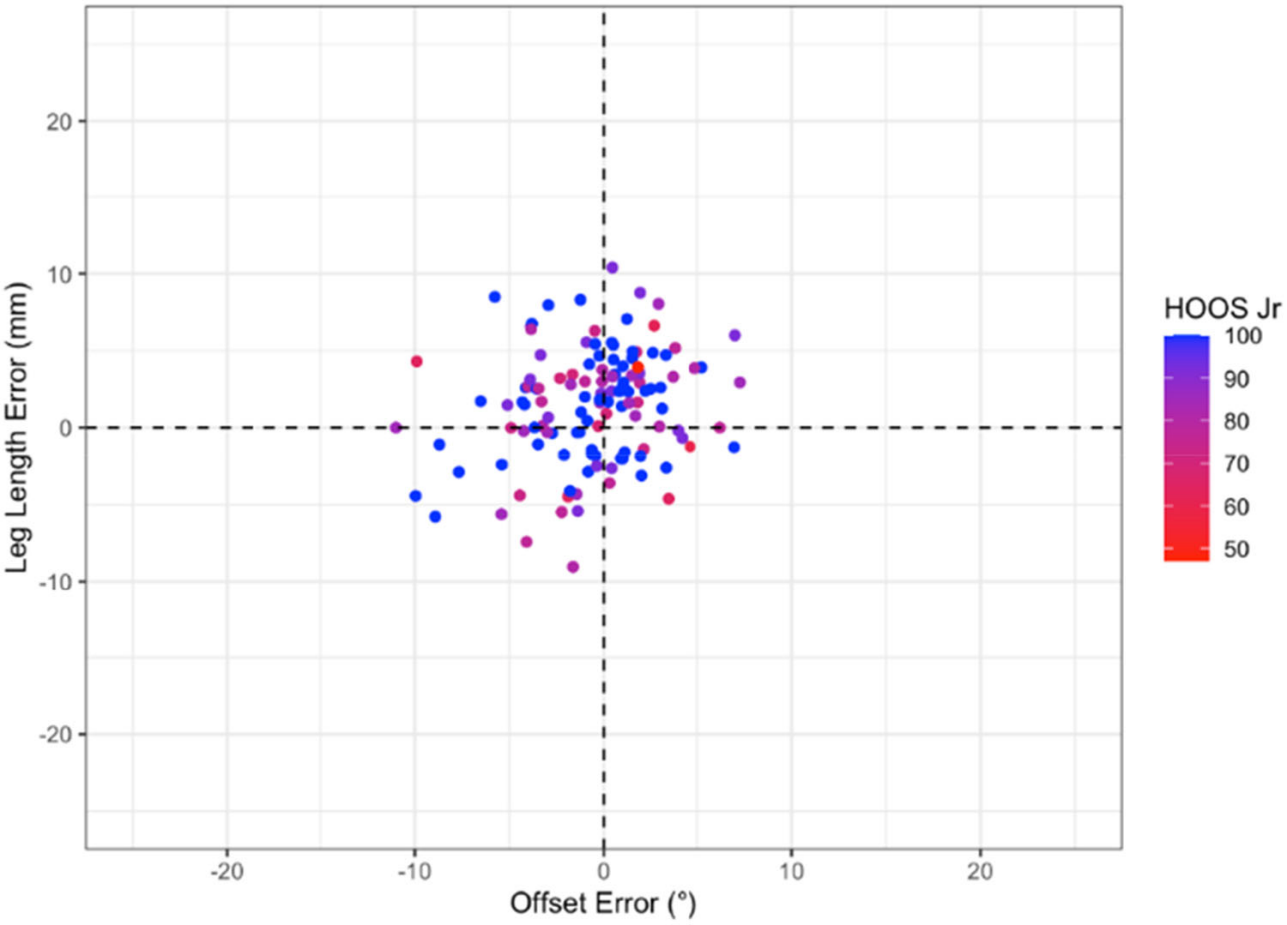
Distribution of HOOS JR according to acetabular cup inclination error and anteversion error (Figure 4) and leg length error and offset error (Figure 5). Leg length and femoral offset are displayed together to reflect their combined influence on hip biomechanics and functional limb symmetry. Progression from red to blue representing higher (better) Oxford Scores. HOOS JR, hip dysfunction and Osteoarthritis Outcome Score for Joint Replacement Score.

There was no significant correlation between the component placement error (leg length, offset, cup inclination and anteversion or combined anteversion) and 12‐month OHS (Pearson range −0.07 to 0.04, *p* > 0.44) or HOOS JR Score (Pearson range −0.15 to −0.01, *p* > 0.09) (Figures 2, 3, 4, 5).

### Complications

Control group: One male patient who suffered a hip dislocation at 4 weeks after THA. This was caused by a fall and so was not thought to be due to component position, and there were no further episodes of instability. One patient had a fracture of the tip of the greater trochanter at 4 weeks postop. This was through a drill hole used to reattach the piriformis and healed uneventfully.

PAR group: One patient experienced loosening of the acetabular component and underwent revision at 9 months, and one patient experienced irritation of her iliopsoas tendon, which was treated with arthroscopic iliopsoas tendon release at 8 months. CT evaluation of both cases showed no acetabular component malposition.

## DISCUSSION

This study demonstrated no significant difference in 12‐month postoperative PROMs between patients who had highly accurate THA implant placement relative to the plan, compared to those with mild errors in component placement. Furthermore, there was no significant correlation between an increasing component placement accuracy and improvement in the OHS or HOOS JR score. Therefore, while highly accurate prosthesis placement is desirable in theory, it did not translate to superior 12‐month PROMs after THA in this study.

Published literature have demonstrated that conventional manual techniques in THA can cause up to 60% of patients to have >10 degrees of variation from their planned acetabular cup placement [24]. Yet, while accuracy has improved with modern assisted techniques including 3D‐printed PSI guides, it has so far been unclear whether this has led to improvements in PROMs. Thomas et al. [28] analysed 64 patients and 12‐month PROMs (OHS and HOOS scores) utilising the OPS^TM^ system including 3D‐printed PSI guides against conventional manual techniques was unable to demonstrate any significant difference. However, the authors also noted that in their study, there was no significant difference in the accuracy identified between the OPS^TM^ and the conventional group, despite the conventional group having a larger distribution of angles, thereby possibly negating any clinical influence that accuracy may have had on PROMs. Moreover, the authors note the possibility that the study was underpowered due to small patient numbers. These results are in contrast to a more recent study by Shoji et al. [26], who identified that combined anteversion on CT, as determined by Dorr et al. [3], femoral stem sagittal alignment and stem rotation had statistically significant effects on ADLs and functional movements such as squatting and deep hip flexion. It is quite possible that these findings are driven by femoral stem placement, with evidence purporting the influence of leg length discrepancy (LLD) and offset in affecting PROMs including pain, as well as gait kinematics [29]. A recent large study found that higher variations in femoral and acetabular offset relative to the native hip on radiographs was associated with poorer HOOS JR scores, however that magnitude of the component placement variations were considerably larger than what was observed in this study [25]. Collectively, this suggests that while large variations outside ideal THA component position may have a significant impact on outcomes, more subtle differences have little effect, supporting the notion that ‘near enough is good enough’.

Postoperative CT evaluation of demonstrated high levels of accuracy using functional alignment and PSI guides when trying to achieve at least one of the PAR elements (Tables 1 and 2). This is in concordance with published literature, with a study by Ferretti et al. [4] and Previ et al. [19] demonstrating similar mean differences of acetabular cup anteversion and inclination utilising the same templating and OPS^TM^ system. Worlicek et al. [29], however, underlined the importance of evaluating the combined variation in both acetabular and femoral implant placement in the implication of post‐operative symptoms including pain including combined anteversion of both implants. Femoral offset is known to be affected by axial rotation, which is difficult to appreciate accurately on anteroposterior radiographs and thus the value of 3D radiological evaluation cannot be understated [13]. Yet despite this notion of combined variation, the PAR group did not show any difference in proportion reporting no hip pain at 12‐months compared to the controls (61% vs. 60%, *p* = 0.871). The lack of difference could be related to the utilisation of a functional alignment philosophy, which aims to recreate a biomechanical environment that is more anatomical while reducing implant or anatomical impingement [21].

Overall, the results demonstrate that 3D‐printed PSI guides can achieve highly accurate implant placement in all three planes relative to preoperative functional alignment templates using the OPS^TM^ system. Yet despite this accuracy, there was no statistically significant relationship between accuracy and 12‐month PROMs across multiples methods of analysis (Figures 1, 2, 3, 4, 5). Thus, it appears that PROMs are inelastic to previously defined radiological margins of implant placement when utilising a functional alignment target.

Despite this, the vast majority of patients are satisfied, reported an improvement, and would have the same surgery again if required, fundamentally highlighting the overall success of THA.

There are several important limitations to this study. First, the retrospective nature of this study did not allow for appropriate a priori sample size selection. However, the group sizes were appropriately powered to detect the minimally important group difference of five points on the OHS [23]. And the observed margin of difference in mean HOOS JR and OHS of only one point suggests that a clinically relevant difference is unlikely. This study analysed 125 patients and future studies evaluate larger populations would improve confidence the findings. Second, the follow‐up period was relative short and thus results identified here cannot be extrapolated into long‐term outcomes particularly in regard to rates of revision from issues such as osteolysis, wear, edge‐loading and impingement. Thirdly, both the PAR group and the control group had a relatively narrow deviation from the planned implant placement. Thus, small errors in component placement are well tolerated, but this cannot be extrapolated to larger errors in placement. Fourthly, there were a higher proportion of patients with a diagnosis of OA in the PAR group, which is a potential confounder. Finally, factors influencing outcomes of THA are multifactorial, encompassing a range of patient, surgical, implant‐related and rehabilitation factors [2], which were not analysed in this study and which could have influenced results.

## CONCLUSION

In conclusion, this study demonstrated no differences in PROMs, including disease specific measures and satisfaction between those with highly accurate THA component placement, compared to those with mild errors in component placement relative to the preoperative plan. No correlation was identified between acetabular and femoral component placement relative to preoperative templates and improvement in PROMs. The implications for hip arthroplasty practice are substantial: achieving high levels of technical accuracy through advanced technologies may not consistently translate into measurable improvements in patient outcomes.

## AUTHOR CONTRIBUTIONS

All authors have made substantial contributions to the conception or design of the work; or the acquisition, analysis or interpretation of data; drafted the work or revised it critically for important intellectual content; approved the version to be published; and agree to be accountable for all aspects of the work in ensuring that questions related to the accuracy or integrity of any part of the work are appropriately investigated and resolved.

## CONFLICTS OF INTEREST STATEMENT

Institutional research support was received from Friends of the Mater Foundation. The senior author receives royalties from Corin UK Pty Ltd., has given paid presentations, and received travel support for meetings from Corin Australia and Depuy Australia, and is the Chairman of the Australian Orthopaedic Association Joint Replacement Registry Committee. GKS is an employee of Corin.

## ETHICS STATEMENT

Approved by St Vincent's Human Research Ethics Committee, Sydney REF 2019/ETH02851. All patients provided informed consent to participation.

## Data Availability

The data associated with our study is not publicly available, in line with our consent and ethics approval process, but specific requests may be sent to the corresponding author.
